# Violence against Afghan women by husbands, mothers-in-law and siblings-in-law/siblings: Risk markers and health consequences in an analysis of the baseline of a randomised controlled trial

**DOI:** 10.1371/journal.pone.0211361

**Published:** 2019-02-07

**Authors:** Rachel Jewkes, Julienne Corboz, Andrew Gibbs

**Affiliations:** 1 Gender & Health Research Unit, South African Medical Research Council, Pretoria, South Africa; 2 Office of the President of the South African Medical Research Council, Pretoria, South Africa; 3 School of Public Health, Faculty of Health Sciences, University of the Witwatersrand, South Africa; 4 Independent Consultant, Kabul, Afghanistan; 5 Centre for Rural Health, School of Nursing and Public Health, University of KwaZulu-Natal, South Africa; Stellenbosch University, SOUTH AFRICA

## Abstract

**Background:**

Violence by mothers-in-law, as well as husbands, is a recognised problem in many countries. It has been given little attention in research on violence and its importance as a health problem, and aggravator of husband violence, has not been well established. Our aim was to describe patterns and the frequency of mother-in-law and sibling-in-law/sibling physical violence in relation to physical violence from husbands, and to describe risk characteristics and associated health behaviours of women with different abuse exposures.

**Methods:**

1,463 women aged 18–48 were recruited into a randomised controlled trial (RCT) to evaluate a women empowerment intervention in 6 villages of Kabul and Nangarhar provinces. The women were interviewed at baseline. The analysis uses bi-variable and multivariable logistic regression.

**Results:**

932 of the women were currently married. Of these, 14% of women experienced mother-in-law physical violence and 23.2% of women experienced physical spousal violence in the previous 12 months. For 7.0% of women, these exposures were combined. Physical violence was associated with food insecurity and having to borrow for food, being in a polygamous marriage, living with their mother-in-law, as well as province of residence (higher in Nangarhar). Women who had earnings were relatively protected. Whilst most mothers-in-law were described in positive terms, those who used physical violence were much less likely to be described so and a quarter were described as very strict and controlling and 16.8% as cruel. Overall slightly more women described their husband in positive terms than their mother-in-law, but there was a very strong correlation between the way in which husbands were perceived and the violence of their mothers.

Women’s mental health (depression, suicidal thoughts and PTSD symptoms score), self-rated general health, disability and beating of their children were all strongly associated with intimate partner violence (IPV) exposure. The strength of the association was much greater for all of these problems if the IPV was combined with physical violence from a mother-in-law or sibling-in-law/sibling. Experienced alone, violence from the mother-in-law or a sibling-in-law/sibling was associated with an elevated risk of all of these problems except depression.

**Interpretation:**

Mother-in-law and sibling-in-law/sibling physical violence is an appreciable problem among the women studied in Afghanistan, linked to poverty. It has a major impact on women’s health, componding the heath impact of IPV. In this setting conceptualising women’s risk and exposure to violence at home as only in terms of IPV is inadequate and the framing of domestic violence much more appropriately captures women’s risks and exposures. We suggest that it may be fruitful for many women to target violence prevention at the domestic unit rather than just at women and their husbands.

## Introduction

Violence against women is recognised as a major impairment to health and social development and it is primarily conceptualised as that perpetrated against a women by a current or ex-husband or boyfriend. This formulation realistically captures women’s primary risk in many global regions, but it does not necessarily do so as well in settings where after marriage women often move into their husband’s parent’s home or set up home with their mother in law. This is a common pattern in many geographical settings, especially those in areas where there is ‘classic patriarchy’ [1], which Kandiyoti described as encompassing North Africa, the Muslim Middle East, and South and East Asia (especially India and China) although it also includes less well documented areas such as Muslim Central Asia, e.g. Tajikistan and Afghanistan. This sets up possibilities for women having a range of other violence exposures within the home in these areas of the world.

Violence by mothers-in-law takes the forms of emotional and physical abuse and has been described in many settings from Mexico to India [2, 3]. Some of the violence mothers-in-law have perpetrated has been described as motivated by jealousy over their son’s affection for his wife and a desire to weaken their bonds [1, 4]. Some by a desire to teach the daughter-in-law where she is located in the family hierarchy and rules of the house, and to demonstrate control over the family [5]. The violence has also been described an expression of power which is wielded by mothers-in-law over their daughters-in-law in order to ensure that they control their daughters-in-laws’ labour [1]. Kandiyoti wrote of the acceptance by young women of this inferior position, stemming from the knowledge that they would accede to the position of greater power when they themselves become mother-in-law [1]. However an assertion that women adopt a subservient position and from that position also justify the prevailing patriarchal gender relations is not tantamount to agreement that there is no harm from experiencing violence in a subordinated role as a young daughter-in-law. The health impact of most forms of violence has been described, but that of mother-in-law violence has been given little attention [6]. Harris has particularly shown that women experience severe isolation from their support networks when they move into their husband’s family home [7]. Social isolation in the face of violence has an important negative impact on women’s mental health. This is often deliberately constructed by discouraging close contact with the natal family [8]. The sense of isolation is compounded when violence is experienced, especially by mothers-in-law and husbands as women are left without support or allies. Conversely women who experience IPV (in countries where there is no or very little dating, this is violence from their husbands) but have mother-in-law support have been shown to be much less severely emotionally effected by the IPV [2].

Erich, in his work in Tajikistan, argues that the term domestic violence better fits the violence experiences of young Tajik women, more than intimate partner or spousal violence [8]. He uses the term advisedly, and “does not seek to demote the importance of patriarchal social norms and structures” (p.32) [8]. Rather, the term allows for inclusion of violence by other in-laws, such as father-in-law and sibling-in-law violence which some women also experience, as well as mother-in-law violence. Erich also described how boys are taught as children to discipline their sisters by beating them if they neglect to perform household chores to the expected standard [8]. This culturally sanctioned violence by male siblings is another dimension of violence perpetrated against women. Erich described domestic violence in Tajikistan as driven by patriarchy and women’s subordinate position in society, and those factors that increased women’s vulnerability increased their likelihood of experiencing violence [8]. Factors increasing women’s vulnerability in marriage included marrying at a young age, having a polygamous marriage and having a religious and not legal marriage. He also described economic vulnerability and dependency of women as a risk factor for in-law violence, specifically in communities that are highly dependent on remittances from migrant workers. Women who received remittances directly from their husbands were less likely to experience in-law violence than those women in families where remittances went to the in-laws. Education only provided protection if women attended secondary school, and abuse only very substantially reduced when women reached over the age of 40 [8].

Very little is known about the prevalence of violence against women by in-laws and other family members in Afghanistan, and how dynamics of intimate partner violence and other family violence intersect at the household level. In one survey in Afghanistan, the husband was the prime abuser for 30.6% of women, but abuse by mothers-in-law was reported by 23.7% [9]. In the recent Afghanistan Demographic and Health Survey, although 94% of ever-married women who had experienced physical violence since the age of 15 reported their current husband as the perpetrator, women also named a range of other perpetrators: 9% reported violence by a mother or stepmother, 8% by a father or stepfather, 7% by a mother-in-law, 7% by a father-in-law and 4% by siblings.[10]

We sought to advance understanding of violence against women in Afghanistan by considering whether ‘domestic violence’ would similarly be a better formulation of the problem, and to describe the health impact of different forms of violence. Interviews with women conducted as the baseline of a randomised controlled trial to evaluation an IPV prevention intervention provided an opportunity to research hypotheses on the occurrence and patterns and impact of mother-in-law, sibling-in-law/sibling and spousal physical violence. The aim of this paper is to describe patterns and the frequency of mother-in-law violence in relation to physical violence from husbands. We hypothesised that these would often, but not always, co-occur. A second aim was to describe risk characteristics and associated health behaviours of women with different abuse exposures. Our second hypothesis was that exposure to spousal violence as well as mother-in-law physical violence would be more strongly associated with ill health and the women’s own use of violence in childrearing, thus impacting exposure to violence of the next generation and fuelling the intergenerational cycle of violence.

## Methods

The interviews were conducted as the baseline for a randomised controlled trial (RCT) evaluation of the women’s economic and social empowerment intervention of the international non-governmental organisation Women For Women International (WFWI). This was part of the What Works To Prevent Violence? A Global Programme on Violence Against Women and Girls (VAWG), which has been funded by the UK Government’s Department For International Development (DFID) to advance the global knowledge on prevention of VAWG. The Global Programme is supporting the evaluation of 11 interventions using rigorous methods to assess their effectiveness in preventing violence against women and girls. This evaluation is part of this overall portfolio of work.

We interviewed 1463 women who lived in Nangarhar and Kabul provinces of Afghanistan. They were recruited from six villages. The sample was thus a volunteer sample of women interested in participation in the intervention, who were then randomised into the two study arms. The eligibility criteria for the trial were being aged 18–45 years, poor (preferably earning less than US$1 per day), able to consent and willing to participate in an intervention held over a year. The villages were selected by WFWI after taking into account a range of geographical, political and social considerations such that the intervention was predicted to be able to be feasibly and safely delivered in these settings. Women were randomised into the intervention or control arm after recruitment into the trial. Control arm women were given US$10 to incentivise each research interview. The number of women interviewed was determined by the sample size calculation for the main RCT.

Interviews were conducted face to face in a private community location (community centre or building serving this purpose) with a female interviewer. Interviewers were women from the provinces in which data was collected. All had completed secondary school and most had a background in teaching. Two were university students at the time of data collection. Most of them had several years’ experience in data collection. A standard questionnaire was developed and translated into Dari and Pashto (with back translation). It was pre-tested with 100 interviews and adjusted after the pre-test. Interviews were conducted with the language of choice for women and responses recorded on paper questionnaires. The questionnaires included questions on social and demographic characteristics of the women, their gender attitudes, perceived community gender attitudes, mental health, sexual and reproductive health and women’s exposure to violence. Most of the measures had established validity in other settings. After pre-testing we considered the responses to items and calculated Cronbach’s alphas to examine internal consistency of scales. We reduced the number of trauma exposures by two, as none of the women reported experiencing these (experience of being kidnapped, robbed at gun point), we deleted an item on whether women had ever attempted suicide, we simplified the contraception questions, and removed two items about the ‘honesty of reporting’ by participants.

Ethical approval for the research was given by the Ethics Committee of the Medical Research Council of South Africa and the Institutional Review Board of the Ministry of Public Health of Afghanistan. As a first stage WFWI connected with the villages and gained support for running the intervention in them. WFWI staff explained that there was to be random selection into the intervention and research conducted. These meetings were initially with community elders and men as well as women so that, as far as possible, women had family support for the research and, if selected, intervention participation. On the day of recruitment, all women interested in participation presented themselves to the site and were screened for trial inclusion. Those screened as eligible were allowed to enter the recruitment rooms where they consented for the study and were randomised into the intervention or control arm. Literacy levels were very low and so women were verbally informed about the research and given a written information sheet. They were asked to give consent on a form with a thumb print. Interviews were conducted on a subsequent day.

### Measures

Physical violence from a husband was assessed with five questions which were taken from the World Health Organisation (WHO)’s instrument [11]. Each item asked about a specific act of violence in the past 12 months, and allowed for never, once, few or many responses, and a binary (ever/never) was created. The questions asked about acts such as whether the woman had been slapped, pushed, hit, threatened with a knife or gun, or had them used on her.

Violence from a mother in-law was asked of women who had met their mother in-law, even if she did not co-reside with them. They were asked if in the ‘last 12 months you were slapped, hit or beaten by your mother-in-law’. Responses were ‘never, sometimes, or often’. Violence from another relative or in-law was asked of all women. They were asked if in the ‘last 12 months they were slapped, hit or beaten by their brother or sister or a brother or sister or other relative of your husband’. Responses were ‘never, sometimes, or often’. These two variables were combined into a single exposure variable of any mother-in-law or sibling-in-law/sibling violence in the past year. Physical violence from the husband and this combined in law violence exposure measure were then combined in a four level dummy variable with the levels: no violence; only in law/sibling violence; husband violence; and both violence exposures. This paper centres the analysis of physical rather than emotional violence chiefly as this is a relatively new area of research and there are no standard questions on physical or emotional by mothers-in-law.

The variable on hitting children was derived from a question asking: “in the last 4 weeks, how often did you punish your children by giving a slap or beating or otherwise physically punishing them?”. It had response options: never, once, 2–3 times and 4 or more times, and was used as a binary variable (ever/never) in logistic regression.

We measured depression using the 20 item CES-D scale [12]. We have dichotomised it at 20/21 scores [13]. To assess suicidality, women were asked if in the past 4 weeks the thought of ending their life had been in their mind. We measured PTSD symptoms using the Harvard Trauma Questionnaire [14]. In the model we used a measure of PTSD symptom mean which was the sum of the 16 items divided by 16. This was done because we did not have the requisite information for PTSD diagnosis (i.e. symptom duration, trauma exposure and frequency). We measured disability using the six item Washington Group scale [15] and developed a dichotomised disability measure whereby women were classes as disabled if they responded that they could not do or had a lot of difficulty with any of the items. A typical item was “Do you have difficulty walking or climbing steps?” To measure general health, women were asked if they would describe their overall health as excellent, good, fair, poor or very poor. This variable was dichotomised as “poor or very poor” versus the other responses.

In addition, we asked about age, highest level of schooling (none, madrassa, primary or secondary) and marital status. If a woman was married we asked about whether her husband had other wives, and if she was married to a cousin or other relative. We assessed poverty by asking three questions about frequency of food insecurity in the last 4 weeks, whether: there had being no food to eat in the house due to lack of money, a member of the household went to sleep without eating due to lack of food, and a member of the household went all day and night without eating due to lack of food. Responses were never, rarely, sometimes and often. These were summed (range 3–12) and a three level variable derived with no (answering ‘no’ to all questions), moderate (score 4–6) and severe food insecurity (responding ‘often’ to one item or scoring overall 7 or more). Women were asked how often in the last four weeks they had had to borrow money or food because they did not have enough, and a dichotomised variable was derived with ‘borrows weekly or more often’ versus ‘less often than weekly or never’ as the two levels. They were also asked if they had done anything to earn money for themselves or their family in the last 3 months.

### Data analysis

Questionnaires were doubled entered into SPSS databases that were merged and verified. Data were analysed in Stata 13.0. Missing data was <5% (for example 3/935 women lack past 12 month IPV data) and imputation was not used. In the analysis we only use women in the trial reporting currently being married. Continuous variables were summarised using means and 95% confidence intervals and categorical variables with percentages and chi squared tests. We used multivariable logistic regression and multiple regression to model the health impact of violence exposure, adjusting for age, education, food insecurity, whether she had earnings and province. We present the adjusted odds ratios or coefficient and 95% confidence intervals and Chi square. We also present the proportion and number of women in each violence exposure category having (or not having) the health problem.

## Results

The prevalence of experience of physical violence in the past 12 months is shown in Table 1. Most of the women (69%) had not experienced physical violence from anyone in the household. 7.8% had experienced it from their mother-in-law or a sibling-in-law/sibling only. 16.2% had experienced it from their husband and 7.0% had experienced it from both. Thus 18.7% of women who had a mother-in-law alive (or who they had met) had experienced past year physical violence from their mother-in-law or a sibling-in-law/sibling, with or without experiencing physical violence from their husband. Physical violence by a sibling-in-law or sibling was common, with overall 11.4% of women experiencing this in the past year and 13.0% had experienced abuse from their mother-in-law in the last year. 5.8% of women had only experienced physical violence from their sibling-in-law/sibling and not from their mother-in-law and 7.4% had experienced physical violence from their mother-in-law and not a sibling-in-law/sibling. 5.6% had experienced both mother-in-law and sibling-in-law/sibling physical violence. Of the women who experienced physical violence from their mother-in-law, 55.5% currently lived with her, 34.6% had previously lived with her and 9.9% reported that she lived elsewhere.

**Table 1:** Prevalence of experience of physical violence in the past 12 months, married women.

	n	%
No violence	643	69.0
Violence from mother-in-law or sibling-in-law	73	7.8
Physical IPV	151	16.2
Both Physical IPV and mother-in-law violence	65	7.0
Total	932	100

Table 2 shows the social, economic and demographic characteristics of the women by their physical violence exposure and tests of association. The mean age of women was 29.28 (range 14–48) and did not differ by violence exposure category. Overall 75.3% of the sample lived in Kabul province and 24.7% in Nangarhar Province. The proportion of women experiencing mother-in-law and sibling-in-law/sibling physical violence in Nangarhar was high, 34.3% for this exposure alone and 46.2% for this exposure combined with husband physical violence. Overall 33.1% of women experience moderate food insecurity and 6.9% experienced severe food insecurity. Both of these were more common in all physical violence exposure categories. Overall 55.9% of women said they had done something to earn money for the family in the last 3 months. Earning was less common among women who experienced mother-in-law and sibling-in-law/sibling violence (45.8% for just this and 47.9% among those also beaten by their husband). Overall 23.2% of women had ever been to school of any form, and nearly a third of these had only been to a madrassa. Thus 16.5% had been to a formal school and only 6.4% had been to junior secondary or high school. The proportion having attended any school did not vary across violence category.

**Table 2:** Characteristics of married women by exposure to domestic violence.

	None n	%	MIL/SIL n	%	p value	IPV n	%	p value	Both n	%	p value
Mean age	643	32.26 (31.69, 32.82)	73	31.48 (29.75, 33.21)	0.388	151	33.26 (32.10, 34.41)	0.129	65	32.82 (31.06, 34.57)	0.556
**Province**:											<0.0001
Kabul	550	85.54	48	65.75		109	72.19		35	53.85	
Nangarhar	93	14.46	25	34.25		42	27.81		30	46.15	
**Food insecurity**:											<0.0001
little/none	439	68.38	39	53.42		62	41.06		20	30.77	
moderate	179	27.88	28	38.36		77	50.99		33	50.77	
severe	24	3.74	6	8.22		12	7.95		12	18.46	
**Earning in past 3 months**											0.003
Yes	405	62.99	33	45.83		98	64.9		31	47.69	
No	238	37.01	39	54.17		53	35.1		34	52.31	
**Education**											0.393
Primary or secondary school	79	12.32	10	13.89		13	8.61		5	7.69	
No school or only madrassa	562	87.68	62	86.11		138	91.39		60	92.31	
**Borrows money for food almost every week or more**											<0.0001
Yes	110	17.13	27	36.99		47	31.33		27	41.54	
No	532	82.87	46	63.01		103	68.67		38	58.46	
**Polygamous marriage**											<0.0001
Yes	29	4.52	3	4.11		14	9.27		17	26.15	
No	613	95.48	70	95.89		137	90.73		48	73.85	
**Married to a cousin or other relative**											0.858
Yes	84	13.1	10	86.11		21	13.91		11	16.92	
No	557	86.9	62	13.98		130	86.09		54	83.08	
**Lives with mother-in-law**											
Currently	182	29	37	51.4		45	30		30	46.2	<0.0001
Previously	203	32.4	24	33.3		32	21.3		16	24.6	
Never co-resided	45	7.2	5	6.9		7	4.7		4	6.15	
Never met her	197	31.4	6	8.3		66	44		15	23.1	

23.0% had borrowed money or food every week or more often, and this was much higher in all the violence exposure categories. Thus 37.0% of women experiencing just mother-in-law and sibling-in-law/sibling physical violence had borrowed often, 31.3% of thus beaten by their husband and 41.5% of those with a combined violence experience. Only 6.8% of the women were in polygamous marriages, and these women were more at risk of physical violence from their spouse and particularly the combined spousal and mother-in-law and sibling-in-law/sibling physical violence (26.2% of this exposure group were polygamously married). Marriage to a cousin or other relative was reported by 13.7% of women. This did not differ much by physical violence exposure category.

Women were much more likely to experience mother-in-law and sibling-in-law/sibling physical violence if they lived with their mother-in-law. Overall 51.4% of women co-residing with their mother-in-law had experience past year mother-in-law and sibling-in-law/sibling physical violence alone, and 46.2% had experienced this combined with husband abuse. In comparison only 29% of women not experiencing physical violence co-resided with their mother-in-law and 30% of those just experiencing physical violence from their husband.

Table 3 shows the overlaps between experience of physical violence by a mother-in-law and a woman’s expressed perceptions of her mother-in-law and her husband. All associations examined were statistically significant. Most women did have positive relationships with their mother-in-law. Nearly two-thirds (63.9%) perceived their mother-in-law understood them, 71.1% perceived their mother-in-law to do be supportive, as far as she could be, 82.2% perceived her to be kind and 78.3% felt she loved her like a daughter. These perceptions were strongly associated with experience of physical violence from the mother-in-law and were less often expressed by those women who had been hit. However very few women indicated that they ‘strongly agreed’ with positive statements. The more qualified response of ‘agree’ and ‘strongly disagree’ was much more often given as a response than ‘strongly agree’, especially to positive statements where the mother-in-law had used physical violence. In all cases woman were much less likely to agree with positive statements about their mother-in-law if they experienced violence.

**Table 3:** Associations between perceptions of her mother-in-law and husband, and abuse from her mother-in-law.

	Strongly disagree %	disagree %	agree %	Strongly agree %	p value		Strongly disagree %	disagree %	agree %	Strongly agree %	p value
My mother-in-law does not really understand me					<0.0001	My husband does not really understand me					0.001
All women	4.0	59.9	33.3	2.9		All women	5.8	68.0	21.4	4.8	
Mother-in law hit her	2.47	33.33	59.26	4.94		Mother-in law hit her	1.23	58.02	38.27	2.47	
did not hit her	4.2	63.8	29.43	2.56		did not hit her	5.9	69.43	19.61	5.05	
My mother-in-law does everything she can to support me					<0.0001	My husband does everything he can to support me					0.001
All women	2.2	26.7	67.4	3.7		All women	1.5	19.3	73.6	5.7	
Mother-in law hit her	8.75	62.5	27.5	1.25		Mother-in law hit her	1.23	37.04	58.02	3.7	
did not hit her	1.28	21.25	73.44	4.03		did not hit her	1.56	17.81	74.85	5.78	
My mother-in-law is a kind person					<0.0001	My husband is a kind person					<0.0001
All women	1.4	16.3	76.5	5.7		All women	1.2	10.4	79.8	8.8	
Mother-in law hit her	6.17	75.31	18.52	0		Mother-in law hit her	1.23	32.1	64.2	2.47	
did not hit her	0.73	7.5	85.19	6.58		did not hit her	0.96	8.29	81.49	9.25	
My mother-in-law loves me like her own daughter					<0.0001	My husband shows he loves me often					<0.0001
All women	2.2	19.5	71.0	7.3		All women	1.5	9.7	77.9	10.8	
Mother-in law hit her	11.11	70.37	17.28	1.23		Mother-in law hit her	2.47	29.63	61.73	6.17	
did not hit her	0.92	11.72	79.12	8.24		did not hit her	1.33	7.86	79.56	11.25	
My mother-in-law is very strict and controlling					<0.0001	My husband is very strict and controlling					0.012
All women	6.3	68.7	23.7	1.3		All women	7.3	69.4	21.4	1.9	
Mother-in law hit her	3.7	29.63	62.96	3.7		Mother-in law hit her	0.00	65.43	32.1	2.47	
did not hit her	6.78	74.54	17.77	0.92		did not hit her	7.33	70.31	20.43	1.92	
My mother-in-law can be cruel					<0.0001	My husband can be cruel					
All women	8.9	74.3	15.7	1.1		All women	8.9	75.6	14.1	1.4	
Mother-in law hit her	2.47	34.57	58.02	4.94		Mother-in law hit her	4.94	61.73	30.86	2.47	<0.0001
did not hit her	9.87	80.26	9.32	0.55		did not hit her	8.66	77.38	12.64	1.32	
My mother-in-law can frighten me					<0.0001	My husband can frighten me					<0.0001
All women	8.9	75.0	15.1	1.1		All women	8.6	75.6	14.5	1.4	
Mother-in law hit her	4.94	32.1	56.79	6.17		Mother-in law hit her	3.7	64.2	28.4	3.7	
did not hit her	9.51	81.35	8.78	0.37		did not hit her	8.53	76.8	13.46	1.2	

A quarter of all women ‘agreed’ or ‘strongly agreed’ that their mother-in-law was very strict and controlling, and among those who had experienced physical violence from her, this was 66.7%. In all 16.8% of women perceived her sometimes to be cruel, and this was 62.9% among those experiencing physical violence from her. A similar percentage (16.2%) reported that their mother-in-law could frighten them. This was 63% among those who had experienced physical violence from their mother-in-law.

Women experiencing physical violence from her mother-in-law were much more likely to agree or strongly agree that their husband did not understand them than other women. Similarly women experiencing this violence were much less likely to perceive that their husband ‘does everything he can to support me’. Most women agreed or strongly agreed that their husband was a kind person, but this was less common if they experienced mother-in-law physical violence. Most women perceived that their husband often showed he loved them, but a third of women (32.1%) experiencing mother-in-law physical violence did not do so. Many women did experience their husband as strict and controlling, but this was more common among women experiencing physical violence from their mother-in-law (34.6% versus 22.4%). Moreover, many women perceived that their husband could be cruel: this was perceived by a third of women (33.3%) experiencing physical violence from their mother-in-law and 14.0% of those who had not. The proportions of women reporting that their husbands could frighten them were similar and showed the same patterns.

Table 4 shows the proportion of women in the physical violence exposure categories experiencing different health problems or beating their children, as well as adjusted associations between the health problem and violence exposure. Overall 25.6% of women were depressed (as determined by CESD 21 or higher), but the proportion ranged from 31.5% (mother-in-law and sibling-in-law/sibling violence only) to 64.6% (mother-in-law and sibling-in-law/sibling and spousal violence) across the physical violence categories. All the odds ratios were elevated but the association was not statistically significantly so for the mother-in-law and sibling-in-law/sibling physical violence exposure alone. The same pattern was seen in an analysis (not shown) using depression as a contonuous variable. The proportion of women reporting suicidal thoughts overall was 6.3%, but this ranged from 9.6% to 41.3% across the physical violence exposure categories with the highest proportion in the combined exposure category and all associations being statistically significant.

**Table 4:** Health status of married women by exposure to domestic violence.

	None n	%/mean	MIL/SIL n	%	IPV n	%	Both n	%
**Depression (CESD 21+)**								
Yes	107	16.67	23	31.51	56	37.09	42	64.62
No	535	83.33	50	68.49	95	62.91	23	35.38
Adjusted association *	ref		1.53 (0.83, 3.86)	0.173	1.87 (1.20, 2.90)	0.005	5.04 (2.73, 9.30)	<0.0001
**Suicidal thoughts in the last 4 weeks**								
Yes	11	1.72	7	9.59	20	13.25	26	41.27
No	628	98.28	66	90.41	131	86.75	37	58.73
Adjusted association	ref		5.00 (1.81, 13.84)	0.002	6.15 (2.79, 13.59)	<0.0001	30.28 (13.22, 69.35)	<0.0001
**General health poor or very poor**								
Yes	77	11.99	16	21.92	48	31.79	24	38.1
No	565	88.01	57	78.08	103	68.21	39	61.9
Adjusted association	ref		1.97 (1.02, 3.82)	0.045	2.58 (1.63, 4.09)	<0.0001	3.37 (1.78, 6.36)	<0.0001
**PTSD (mean score of symptoms)**	643	1.44	73	1.67	151	1.75	65	2.04
Adjusted association	ref		0.114 (0.013, 0.215)	0.03	0.164 (0.09, 0.239)	<0.0001	0.347 (0.239, 0.455)	<0.0001
**Beat kids in last 4 weeks**								
Yes	240	42.93	49	73.13	110	84.62	49	84.48
No	319	57.07	18	26.87	20	15.38	9	15.52
Adjusted association	ref		3.02 (1.68, 5.43)	<0.0001	5.82 (3.47, 9.77)	<0.0001	5.32 (2.49, 11.36)	<0.0001
**Disability**								
Yes	189	29.62	36	49.32	87	58.39	42	64.62
No	449	70.38	37	50.68	62	41.61	23	35.38
Adjusted association	ref		2.08 (1.22, 3.56)	0.007	2.49 (1.67, 3.70)	<0.0001	2.75 (1.54, 4.93)	0.001

* adjusted for age, education, food insecurity, earning, province, other trauma exposure, redidence with mother-in-law.

Overall 16.4% of women rated their health as poor or very poor, and this ranged from 21.9% (mother-in-law and sibling-in-law/sibling violence) to 38.1% (combined violence exposure) across the phyiscal violence categories, with all associations being statistically significant. The mean score for PTSD symptoms overall was 1.54. This was significantly higher for all physical violence exposures, when compared to the unexposed group. Overall 33.9% of women were classified as having a disability, but the proportion ranged from 49.3% (mother-in-law and sibling-in-law/sibling violence) to 64.6% (combined exposure) across the categories. For every assessed health outcome, the proportion of affected women was higher among women experiencing combined mother-in-law and sibling-in-law/sibling physical violence and husband abuse, than it was among the women who just experienced physical IPV.

Overall 54.0% of women had slapped, beaten or otherwise physically punished one of their children in the last 4 weeks. This proportion was much higher among women who had themselves experienced physical violence in the past year. The proportion ranged from 73.1% (mother-in-law and sibling-in-law/sibling physical violence) to 84.5/84.6% (categories including husband abuse). This was the only outcome assessed where there was no difference between prevalence in the combined category and the category of just being exposed to husband physical violence.

## Discussion

We have shown that both physical violence from the husband and mother-in-law and sibling-in-law/sibling are substantial problems in the lives of many Afghan women in the study sample. There is a very strong association between the way women are treated by the husbands and by their mother-in-law, but it is not a complete overlap. Co-residence with the mother-in-law is clearly a factor for mother-in-law physical violence, but we have shown that this does not always result in mother-in-law violence, nor is it always a necessary component for women to experience this abuse.

We found that mother-in-law and sibling-in-law/sibling physical violence was highest in Nangarhar province, which reflects a pattern of all forms of violence being more often reported by women living here [16]. It was highest in families with more food insecurity, where women had not earned in the last 3 months, borrowed money or food frequently, and were polygamously married. These are all factors which were described by Erich in Tajikistan [8] and are also similar risk factors for women’s experience of physical IPV from husbands in Afghanistan [17].

For all the health outcomes studied, mother-in-law and sibling-in-law/sibling physical violence elevated the prevalence of the health outcome and was remained associated with the health outcome after adjustment for key social, ecponomic and demographic variables. Husband physical violence was even more strongly associated with the health outcome, but the women who were exposed to the combined forms of violence were the most affected. This demonstrates the importance of mother-in-law and sibling-in-law/sibling violence as an adverse exposure for women and strongly suggests that it should be taken seriously and addressed in interventions to prevent violence against women in key areas where it is a substantial phenomenon.

Although the prevalence of mother-in-law and sibling-in-law/sibling physical violence in our study was a little lower than that found in the Global Rights study [9], our measures were of recent exposure to this form of violence. The very poor mental and physical health outcomes found among the women experiencing combined abuse, provides strong support for Harris’s assertion that isolation of women is compunded when women are not supported by their mothers-in-law, as well as evidence that exposure to all forms of violence is harmful and in itself isolating for women[7].

Mother-in-law and sibling-in-law/sibling physical violence is a significant problem for the women studied in Afghanistan and one would expect this to be the case more widely in the ‘mother-in-law belt’[18]. Krishnan[2] has hypothesised that mothers-in-law can also be allies for women who experience violence from their husband, but our findings do point to them being independently violent or co-abusers. These findings suggest that mother-in-laws may contribute to a culture of violence in the home and a wider perception of of entrapment of women in their home, that exacerbates the health impact of the individual violent acts. It is also possible that frequency and severity of violence are worse for women with a violent mother-in-law and that she may also instigate partner violence. [19, 20]

We have also shown that women with a physically violent mother-in-law, like those with a violent husband [21], beat their children more often (as judged by more beating in the last 4 weeks). This increases the children’s risk of IPV experience and perpetration in later life and shapes their views on the normative nature of violence [22, 23].

If we correctly surmise that within families there is a ‘culture’ of violence and a clustering of different forms of violence in a household, it is important to develop and test interventions to prevent “domestic violence” in this setting rather than just “husband violence”. An example of such an intervention is the Zindagii Shoista intervention evaluated among families in Tajikistan [24]. This is a combined gender empowerment and income generating activity, focused on the family, rather than individual women. The association between mother-in-law and sibling-in-law/sibling physical violence and poverty supports the value of this type of approach, as well as tackling gender norms at the family, rather than the individual level.

This study had some limitations. We have focused on physical violence as we did not have a measure of emotional abuse by mother-in-law and sibling-in-law/sibling, and we did not have a measue of husband’s sexual violence perpetration as it was considered too sensitive a question to ask about in Afghanistan. We asked a joint question about violence experienced from a sibling-in-law or sibling and so cannot distinguish these two. We did not ask about the sex of siblings. We did not ask about violence by fathers-in-law and other family members as this is much less often mentioned in this region than mothers-in-law violence and had not been raised by participants in our qualitative research. This may be because of the substantial gender-segregation in daily life. We would have liked to have been able to model mother-in-law violence separately from sibling-in-law/sibling violence but did not have enough sample size to do this. The study was a baseline for a trial and so does not have population-level prevalence measures. Women were not randomly selected but they were not selected for the study based on their exposure to violence. The study was cross-sectional and so we cannot always be sure about the temporal sequence of events, although both the violence exposures and the main health outcomes were contempoareous. Ideally longitudinal research would be of value. We also cannot be sure that mother-in-law and sibling-in-law/sibling violence and husband abuse were not under-reported as women are very closely supervised by their mothers-in-law when they lived with them and many were brought by mothers-in-law to the research recruitment centres. This may have intimidated women when they answered questions, however the effect of under-reporting would be to bias towards the null, which is not evidently a major problem. We did not ask about physical violence used by women against their husbands or their mothers-in-law as this has not been raised as a particular problem in the study area.

## Conclusions

Mother-in-law and sibling-in-law/sibling physical violence is an appreciable problem among the women studied in Afghanistan. It was linked to poverty, and one suspects conflict over scarce resources, at home. We have shown it to have major impact on women’s health and componds the heath impact of husband’s violence. In this setting conceptualising women’s risk and exposure to violence at home as only in terms of IPV, as a dyadic construct, is inadequate and the framing of domestic violence much more appropriately captures women’s risks and exposures. We suggest that it may be fruitful for many women to target violence prevention at the domestic unit rather than just at women and their husbands. Further research is needed on mother-in-law and sibling-in-law/sibling violence in Asia.
